# Dynamic Targetable Extracellular Vesicle Surface Proteins Monitor Depth of Response to CAR T Therapy

**DOI:** 10.21203/rs.3.rs-8913641/v1

**Published:** 2026-03-18

**Authors:** Chen Zhao, Lei Qiu, Ning An, Yong Ju, Jacqueline Ziqian Yang, Hyoyong Kim, Ryan Y. Zhang, Junseok Lee, Jingjing He, Liang-Yan Chen, Yaya Xu, Yue Ma, Emily Ren, Sophie Pan, Pranay Sashikanth, Jina Kim, Di Wang, Peiling Zhang, Jianlin Hu, Yang Gao, Yuhan Bao, Yanhua Tu, Li Wang, Zhili Wang, Zhicheng Zhang, Renjun Pei, Edwin M. Posadas, Sungyong You, Wanxing Chai-Ho, Tasha L. Lin, Sarah M. Larson, Dinesh S. Rao, Hsian-Rong Tseng, Na Sun, Chunrui Li, Yazhen Zhu

**Affiliations:** 1 Department of Pathology and Laboratory Medicine, David Geffen School of Medicine, University of California, Los Angeles (UCLA), Los Angeles, CA 90095, USA; 2 California NanoSystems Institute, Crump Institute for Molecular Imaging, Department of Molecular and Medical Pharmacology, University of California, Los Angeles, CA 90095, USA; 3 Cancer Center, Renmin Hospital of Wuhan University, Wuhan 430060, China; 4 Jiangsu Key Laboratory of Organoid Engineering and Precision Medicine, Suzhou Institute of Nano-Tech and Nano-Bionics, Chinese Academy of Sciences, Suzhou 215123, China; 5 School of Nano-Tech and Nano-Bionics, University of Science and Technology of China, Hefei 230026, China; 6 Department of Hematology, Tongji Hospital of Tongji Medical College, Huazhong University of Science and Technology, Wuhan 430030, China; 7 Division of Cancer Biology and Therapeutics, Departments of Surgery, Cedars-Sinai Medical Center, Los Angeles, CA 90048, USA; 8 Nanjing IASO Medical Technology Co. Ltd., Nanjing 210018, China; 9 Department of Gastroenterology, Institute of Liver and Gastrointestinal Diseases, Hubei Key Laboratory of Hepato-Pancreato-Biliary Diseases, Tongji Hospital of Tongji Medical College, Huazhong University of Science and Technology, Wuhan 430030, China; 10 Division of Medical Oncology, Department of Medicine, Cedars-Sinai Medical Center, Los Angeles, CA 90048, USA; 11 Division of Hematology and Oncology, Department of Medicine, David Geffen School of Medicine, University of California, Los Angeles (UCLA), Los Angeles, CA 90095, USA; 12 Broad Stem Cell Research Center, University of California, Los Angeles (UCLA), Los Angeles, CA 90095, USA; 13 Jonsson Comprehensive Cancer Center, University of California, Los Angeles (UCLA), Los Angeles, CA 90095, USA; 14 Key Laboratory of Vascular Aging, Ministry of Education, Tongji Hospital, Tongji Medical College, Huazhong University of Science and Technology, Wuhan 430030, China; 15 Immunotherapy Research Center for Hematologic Diseases of Hubei Province, Wuhan, Hubei 430030, China

## Abstract

Extracellular vesicles (EVs) represent a promising liquid biopsy platform in multiple myeloma (MM). We developed an MM EV Surface Protein Assay to quantify and dynamically monitor four MM EV subpopulations defined by targetable MM surface proteins (BCMA, CD38, GPRC5D, and CD319) across 336 serial blood samples from 45 relapsed/refractory MM (RRMM) patients treated with anti-BCMA chimeric antigen receptor (CAR) T-cell therapy. All four MM EV subpopulations significantly decreased in 43 patients with initial response, while BCMA^+^, GPRC5D^+^, and CD319^+^ MM EVs increased in 19 patients with progression, and antigen escape was detected by BCMA^+^ MM EVs. MM EV subpopulations differentiated minimal residual disease (MRD) status and complemented MRD for detecting early relapse before clinical progression. Notably, CD319^+^ MM EVs were early predictors of progression-free and overall survival in MRD-negative patients. This assay enables noninvasive monitoring of deep response, progression, and antigen escape, and stratifies survival in MRD-negative patients with RRMM.

## Introduction

Multiple myeloma (MM) is a plasma-cell neoplasm that accounts for approximately 10% of all hematologic malignancies, with an increasing incidence worldwide ^1^. Although therapeutic advances have improved initial treatment responses, most patients eventually develop relapsed/refractory MM (RRMM) ^2,3^. Among the emerging immunotherapies targeting MM-specific surface proteins^4^, such as chimeric antigen receptor (CAR) T cells ^5,6^, bispecific antibodies ^7,8^, antibody drug conjugates ^9^, and monoclonal antibodies ^10^, CAR-T cell therapies targeting B-cell maturation antigen (BCMA) have demonstrated remarkable efficacy in patients with heavily pretreated RRMM, leading to the regulatory approval of several anti-BCMA CAR T-cell therapies, including Idecabtagene vicleucel ^5^, Ciltacabtagene autoleucel ^11^, Zevorcabtagene autoleucel ^12^, and Equecabtagene autoleucel ^13^. Despite these advances, many RRMM patients ultimately experience relapse or disease progression even after achieving deep responses, as indicated by minimal residual disease (MRD) negativity ^14^. Relapse is often driven by antigen escape or even loss of targetable surface proteins ^15,16^. Therefore, in addition to dynamically monitoring depth of response and disease progression, tracking the evolution of targetable surface proteins remains critical to guide salvage treatment strategies in RRMM patients receiving CAR T-cell therapies ^17^.

MRD status is increasingly recognized as an early endpoint for evaluating treatment response and long-term clinical outcomes ^18,19^. Currently protocols rely on invasive bone marrow aspiration to assess MRD via next-generation flow cytometry (NGF) or next-generation sequencing (NGS). Similarly, targetable surface protein profiling in progressing RRMM patients also relies primarily on invasive bone marrow aspirations ^20^, followed by quantification and phenotyping via NGF or immunohistochemistry (IHC) ^21,22^. Although loss of BCMA confirmed by NGF and IHC in biopsied bone marrow has been recognized as a mechanism of therapeutic resistance after anti-BCMA CAR T-cell therapy ^5,23^, the invasiveness of repeated bone marrow sampling limit the frequency of assessment, thereby constraining dynamic detection of both MRD status and targetable MM surface proteins ^24^. Liquid biopsy strategies, such as circulating tumor cell (CTC) analysis, offer a noninvasively alternative ^25^ but are constrained by variable sensitivity and lack of standardization across platforms ^26^. Furthermore, circulating tumor DNA (ctDNA)-based assays have been developed for monitoring MRD ^27,28^, treatment response ^29,30^, and survival ^31^ in MM, they often require patient-specific sequencing panels based on matched bone marrow samples ^22,31,32^ and are not capable of directly measuring surface proteins. As such, ctDNA assays have limited utility in tracking the immunotherapy antigen escape or identifying alternative targetable surface proteins.

In contrast, extracellular vesicles (EVs) have emerged as a promising liquid biopsy platform in MM ^33–36^. These phospholipid bilayer-enclosed particles are secreted by virtually all cell types ^37,38^, with tumor-derived EVs being released at markedly higher levels during disease progression. Importantly, tumor-derived EVs retain tumor-associated surface proteins on their membranes and encapsulate molecular cargo, enabling dynamic assessment of targetable surface proteins in RRMM patients receiving CAR T-cell therapies. Dynamic quantification of these surface proteins on MM EVs may thus allow real-time monitoring of both therapeutic antigen escape and the emergence of alternative targetable antigens ^38–40^. Leveraging these biological characteristics, our recent studies ^41–43^ demonstrated a streamlined EV Surface Protein Assay that integrates click chemistry-mediated tumor EV enrichment and RT-qPCR quantification of multiple tumor EV subpopulations in parallel. Therefore, we hypothesized that this platform could be applied to quantify multiple MM EV subpopulations defined by targetable MM surface proteins over multiple follow-up time points. This approach may inform the evolution of targetable MM surface proteins while enabling dynamic monitoring of the depth of treatment response and prediction of survival after CAR T-cell therapy.

**Fig. 1.**
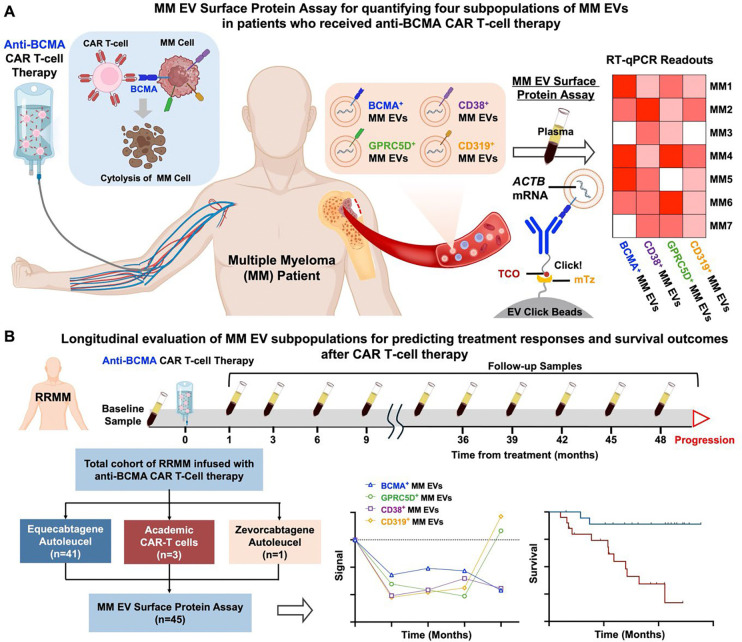
Multiple myeloma (MM) extracellular vesicle (EV) Surface Protein Assay for monitoring treatment response and predicting survival outcomes in MRD-negative patients with RRMM undergoing anti-BCMA CAR T-cell therapy. **(A)** Schematic illustration of the MM EV Surface Protein Assay workflow. Patients with RRMM received a single intravenous infusion of anti-BCMA CAR T-cells following lymphodepleting chemotherapy. Four plasma-derived MM EV subpopulations (i.e. BCMA^+^ MM EVs, CD38^+^ MM EVs, GPRC5D^+^ MM EVs, and CD319^+^ MM EVs) expressing clinically targetable surface proteins were quantified by the two-step assay via i) Click chemistry-mediated enrichment of MM EVs by EV Click Beads in the presence of one of the four trans-cyclooctene (TCO)-grafted antibodies specific to targetable MM surface proteins, and ii) quantification of enriched MM EVs by reverse transcription-quantitative polymerase chain reaction (RT-qPCR) detecting the *ACTB* mRNA within MM EVs. **(B)** Longitudinal quantification of MM EV subpopulations for monitoring treatment responses and predicting survival outcomes after CAR T-cell therapy. A total of 336 plasma samples were collected from 45 patients with RRMM at baseline and multiple follow-up time points after CAR T-cell therapy. MM EV Surface Protein Assay was performed to track the dynamic evolution of the four MM EV subpopulations. The resulting MM EV signals reflect the evolution of the four targetable MM surface proteins and correlate with disease progression, treatment response, minimal residual disease (MRD) status, progression-free survival (PFS), and overall survival (OS). BCMA, B-cell maturation antigen; CAR, chimeric antigen receptor; CD, cluster of differentiation; EV, extracellular vesicle; GPRC5D, G protein-coupled receptor family C group 5 member D; MM, multiple myeloma; mTz, methyltetrazine; RRMM, relapsed/refractory multiple myeloma; RT-qPCR, reverse transcription-quantitative polymerase chain reaction; TCO, trans-cyclooctene.

In this study, we collected a total of 336 longitudinal blood samples from 45 patients with RRMM who received anti-BCMA CAR T-cell therapy. All plasma samples were subjected to quantification of the four MM EV subpopulations (i.e. BCMA^+^, CD38^+^, GPRC5D^+^, and CD319^+^ MM EVs), each defined by a targetable MM surface protein using the MM EV Surface Protein Assay (Fig. 1A). The four targetable MM EV surface proteins were rigorously selected through a bioinformatics framework that integrated established immunotherapy targets from clinical trial databases with multi-omics data, and subsequently validated in MM cell lines, cell-derived EVs, and clinical sample from 30 MM patients versus 30 healthy donor (HD). Our findings demonstrated that dynamic changes of MM EV subpopulations enable noninvasive monitoring of treatment response, disease progression and antigen escape. Notably, MM EV subpopulations also differentiate MRD status and complement MRD assessment for detecting depth of response and early relapse, and further stratify survival in MRD-negative patients.

## Results

### Patient characteristics

From 2020 to 2024, 47 consecutive patients with RRMM who received anti-BCMA CAR T-cell therapy were assessed for eligibility. Among them, 45 patients met the inclusion criteria and entered the study (Fig. 1B, detailed in **Supplementary Methods**). Table 1 outlines the characteristics of patients with RRMM. The median age of the patients was 57 years (range, 53–61), whereas 51.1% (n = 23) of the patients were females and 48.9% (n = 22) were males. According to the Revised International Staging System (R-ISS), 7 (16.3%) patients had R-ISS-1, 30 (69.8%) had R-ISS-2, and 6 (13.9%) with R-ISS-3. In addition, 10 (23.3%) patients had one high-risk cytogenetic abnormality, and 4 patients (9.3%) presented with two high-risk cytogenetic abnormalities.

### Analytical Study of MM EV Surface Protein Assay for Quantifying the Four MM EV Subpopulations

**Fig. 2.**
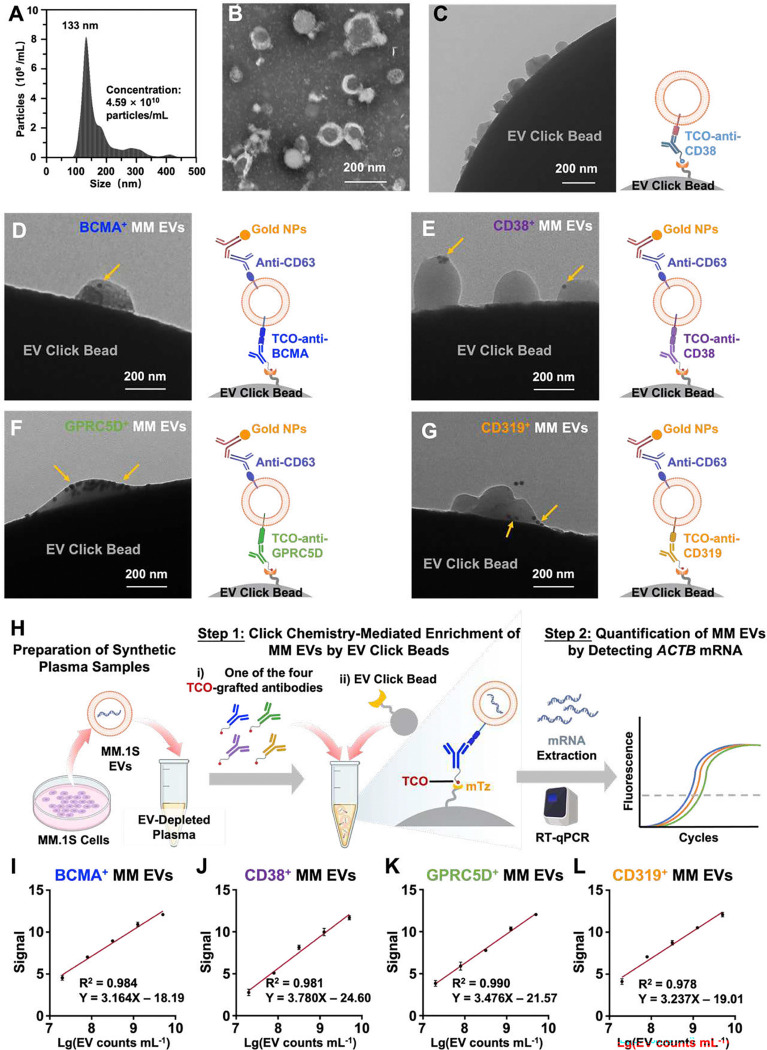
Characterization of MM.1S EVs enriched by EV Click Beads and linearity study of MM EV Surface Protein Assay using synthetic plasma samples. **(A)** Size distribution of MM.1S EVs based on nanoparticle tracking analysis (NTA). **(B)** A representative transmission electron microscopy (TEM) image of MM.1S EVs, revealing their cupped or spherical morphologies for MM.1S EVs. Scale bar, 200 nm. **(C)** A representative TEM image of EV Click Beads with immobilized MM.1S EVs using TCO-anti-CD38. Scale bar, 200 nm. **(D-G)** Representative TEM images of MM.1S EVs enriched on an EV Click Bead using TCO-anti-BCMA (D), TCO-anti-CD38 (E), TCO-anti-GPRC5D (F), and TCO-anti-CD319 (G), followed by immunogold staining with anti-CD63-grafted gold nanoparticles (gold arrows). After click chemistry mediated immobilization of TCO-anti-BCMA, TCO-anti-CD38, TCO-anti-GPRC5D, and TCO-anti-CD319 labelled MM.1S EVs, immunogold staining targeting CD63 (a representative EV surface marker) was performed to further validate the identity of the enriched EVs. Scale bar: 200 nm. **(H)** A schematic illustration of the workflow developed for linearity study of MM EV Surface Protein Assay using synthetic plasma samples. Synthetic plasma samples were prepared by serially spiking MM.1S EVs into EV-depleted healthy donor (HD) plasma. Then the two-step MM EV Surface Protein Assay was carried out. **(I-L)** Dynamic linearity ranges of *ACTB* mRNA signals observed for the four subpopulations of MM EVs enriched by TCO-grafted MM EV-specific antibodies, i.e., TCO-anti-BCMA (I), TCO-anti-CD38 (J), TCO-anti-GPRC5D (K), and TCO-anti-CD319 (L). A strong linear correlation (R^2^ ranging from 0.978 to 0.990) between *ACTB* signal and spiked MM.1S EV concentrations (ranging from 4.0 × 10^6^ to 4.0 × 10^9^ EVs/mL) was observed. Signal was defined as 40 – Ct value. Values are the mean of three replicates. The data show mean ± SD. BCMA, B-cell maturation antigen; CD, cluster of differentiation; EV, extracellular vesicle; GPRC5D, G protein-coupled receptor family C group 5 member D; MM, multiple myeloma; mTz, methyltetrazine; RT-qPCR, reverse transcription-quantitative polymerase chain reaction; TCO, trans-cyclooctene.

MM.1S EVs were characterized through nanoparticle tracking analysis (NTA), transmission electron microscopy (TEM) and scanning electron microscopy (SEM). NTA indicated a consistent average size of 133.0 ± 66.9 nm (Fig. 2A), and TEM and SEM micrographs revealed their cupped or spherical morphologies for MM.1S EVs (Fig. 2B–C). Through an integrated bioinformatic framework (see **Supplementary Methods**), four targetable MM EV surface proteins (i.e. BCMA, CD38, GPRC5D, and CD319) were selected to enrich MM EVs (**Fig. S1**). Expression of all four proteins was validated in the MM.1S cell line (**Fig. S2**). Immunogold staining and TEM were then used to confirm the expression of these four targetable MM EV surface protein markers on MM EVs. After click chemistry-mediated immobilization of TCO-anti-BCMA, TCO-anti-CD38, TCO-anti-GPRC5D, and TCO-anti-CD319 labelled MM.1S EVs, immunogold staining targeting CD63 (a representative EV surface marker) was performed to further validate the identity of the enriched EVs. The results confirmed the presence of the four targetable MM EV surface protein markers on MM.1S EVs and demonstrated successful immobilization of enriched EVs on the EV Click Beads (Fig. 2D–G). We then assessed the linearity of the MM EV Surface Protein Assay by using synthetic plasma samples containing serially diluted MM.1S EVs (Fig. 2H). A strong linear correlation (R^2^ ranging from 0.978 to 0.990) between *ACTB* signal and spiked MM.1S EV concentrations (ranging from 4.0 × 10^6^ to 4.0 × 10^9^ EVs/mL) was observed (Fig. 2I–L), demonstrating excellent linearity and supporting the quantitative robustness of the assay.

**Fig. 3.**
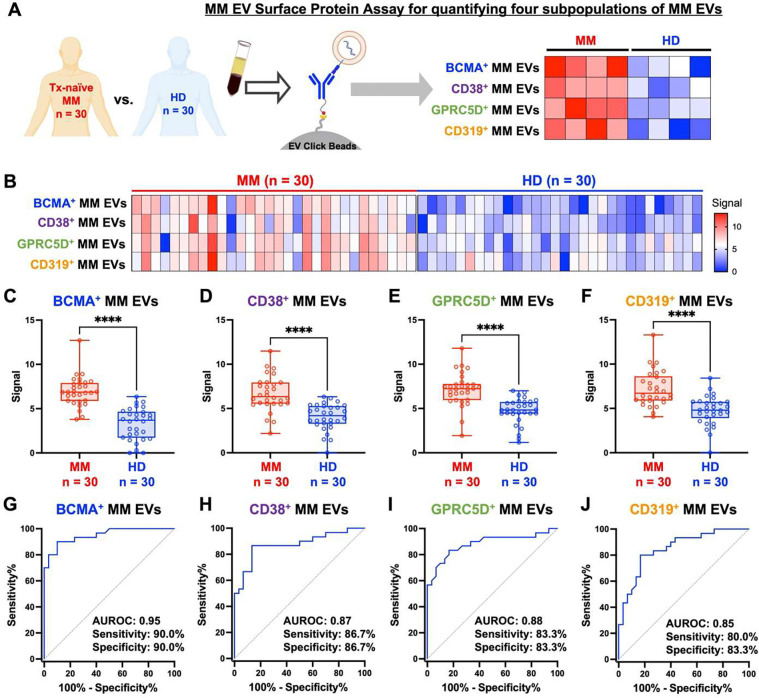
Analytical study of MM EV Surface Protein Assay to distinguish MM patients from healthy donors (HDs). **(A)** A general workflow illustrating the analytical study of MM EV Surface Protein Assay using clinical plasma samples for distinguishing treatment-naive MM patients (n = 30) from healthy donors (HDs) (n = 30). **(B)** A heatmap summarizing *ACTB* mRNA signals in the four subpopulations of MM EVs, including BCMA^+^, CD38^+^, GPRC5D^+^, and CD319^+^ MM EVs. **(C-F)** Significantly higher signals of BCMA^+^ MM EVs (C), CD38^+^ MM EVs (D), GPRC5D^+^ MM EVs (E), and CD319^+^ MM EVs (F) were observed in MM patients (n = 30) compared to those in HDs (n = 30). The signal is represented as 40 – Ct value. The data shows the mean ± SD, and the statistical significance was compared using the unpaired Student’s t-test. **(G-J)** ROC curves of BCMA^+^ MM EVs (G), CD38^+^ MM EVs (H), GPRC5D^+^ MM EVs (I), and CD319^+^ MM EVs (J) for distinguishing MM patients (n = 30) from HDs (n = 30). Significant differences were expressed as: ****(p <0.0001). AUROC, area under receiver operating characteristic curve; BCMA, B-cell maturation antigen; CD, cluster of differentiation; EV, extracellular vesicle; GPRC5D, G protein-coupled receptor family C group 5 member D; MM, multiple myeloma.

To evaluate the performance of MM EV Surface Protein Assay for quantifying the four MM EV subpopulations (i.e., BCMA^+^, CD38^+^, GPRC5D^+^, and CD319^+^ MM EVs), we carried out an analytical study (Fig. 3A) comparing treatment-naïve MM patients to HDs. A total of 60 plasma samples were collected from 30 patients with treatment-naïve MM and 30 HD (Demographic and clinical characteristics of the participants are provided in **Table S1**). For each participant, 400 μL of plasma was subjected to the two-step MM EV Surface Protein Assay that quantifies the four MM EV subpopulations (Fig. 3B). Significantly elevated signals in each MM EV subpopulation were detected in the MM group compared to the HD group (Fig. 3C–F). The diagnostic performance of four subpopulations of MM EVs was summarized in Fig. 3G–J, with the area under receiver operating characteristic curve (AUROC) ranging from 0.85 to 0.95. These results indicate that the MM EV Surface Protein Assay can effectively quantify the four MM EV subpopulations and differentiate MM patients from HD.

### Dynamic Changes of MM EV Subpopulations Reflect Evolution of Four Targetable Surface Proteins in RRMM Patients after Anti-BCMA CAR T-Cell Therapy

**Fig. 4.**
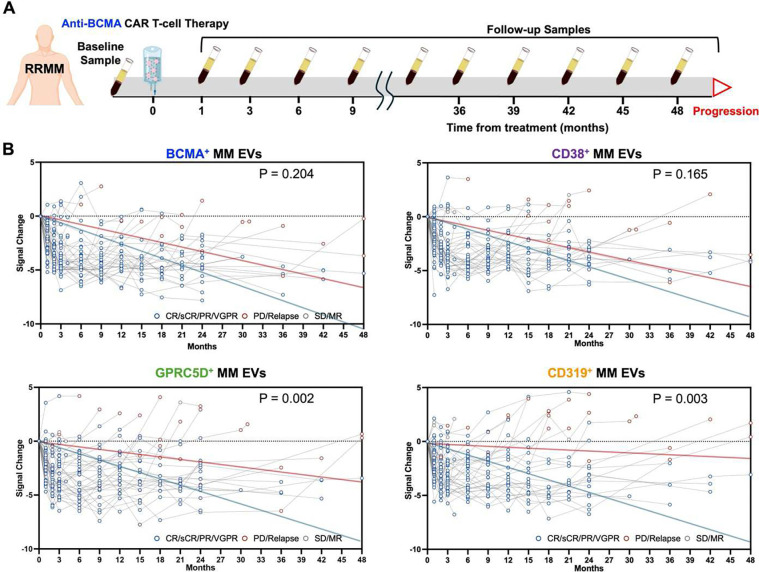
Dynamic monitoring of four MM EV subpopulations by MM EV Surface Protein Assay in RRMM patients (n = 45) who received anti-BCMA CAR T-cell therapy. **(A)** Schematic timeline depicting blood sample collection at baseline and multiple follow-up time points after anti-BCMA CAR T-cell therapy until progression, with longitudinal monitoring using the MM EV Surface Protein Assay. **(B)** Spaghetti plots summarizing the longitudinal signal changes of four MM EV subpopulations (i.e., BCMA^+^ MM EVs, CD38^+^ MM EVs, GPRC5D^+^ MM EVs, and CD319^+^ MM EVs) in each RRMM patient over a follow-up period of up to 48 months. The signal change was calculated for each patient by subtracting their baseline concentration values from follow-up measurements. The red and blue lines illustrate signal trends within the disease progression (PD/relapse) and treatment response (CR/sCR/PR/VGPR) groups, respectively. Differences in the slopes of the regression lines were assessed using Wald t-tests. BCMA, B-cell maturation antigen; CAR, chimeric antigen receptor; CD, cluster of differentiation; CR, complete response; EV, extracellular vesicle; GPRC5D, G protein-coupled receptor family C group 5 member D; MM, multiple myeloma; MR, minor response; PD, progressive disease; PR, partial response; RRMM, relapsed/refractory multiple myeloma; sCR, stringent complete response; VGPR, very good partial response.

We monitored the dynamic changes of the four MM EV subpopulations in 45 RRMM patients after anti-BCMA CAR T-Cell therapy. Plasma samples were collected at multiple follow-up time points until progression and analyzed using the MM EV Surface Protein Assay (Fig. 4A). For each patient, signal changes of the four MM EV subpopulations were calculated by subtracting baseline signals from their signals at follow-up time points. Complete or partial response was defined according to IMWG criteria as the time point at which a patient achieved partial response (PR), very good partial response (VGPR), complete response (CR), or stringent complete response (sCR). Disease progression was defined by IMWG criteria as PD or relapse after CR. Longitudinal data were visualized using spaghetti plots to examine individual trajectories (Fig. 4B). Signal changes in four MM EV subpopulations decreased in patients achieving complete or partial response (CR/sCR/PR/VGPR), whereas signal changes increased in patients who experienced progressive disease (PD/relapse). To statistically evaluate these longitudinal patterns, we applied linear mixed-effects models and compared the slopes of fitted regression lines between response and progression groups using Wald t-tests. Among the four MM EV subpopulations, GPRC5D^+^ MM EVs and CD319^+^ MM EVs exhibited significantly different slopes between two groups (P = 0.002 an P = 0.003, respectively). These results indicate that dynamic changes in specific MM EV subpopulations quantitively reflect treatment response of patients with RRMM after receiving anti-BCMA CAR T-cell therapy.

### MM EV Subpopulations Detect Treatment Response and Progression, and Reflect BCMA Antigen Escape Following Anti-BCMA CAR T-Cell Therapy

To evaluate whether MM EV subpopulations can detect treatment responses and disease progression in RRMM patients following anti-BCMA CAR T-cell therapy, we first compared the signals of four MM EV subpopulations between baseline and the first documented response, and between the first documented response and the first documented progression. The first documented response was defined according to IMWG criteria as the earliest time point at which a patient achieved PR, VGPR, CR, or sCR. The first documented disease progression was defined by IMWG criteria as PD or relapse after CR.

**Fig 5.**
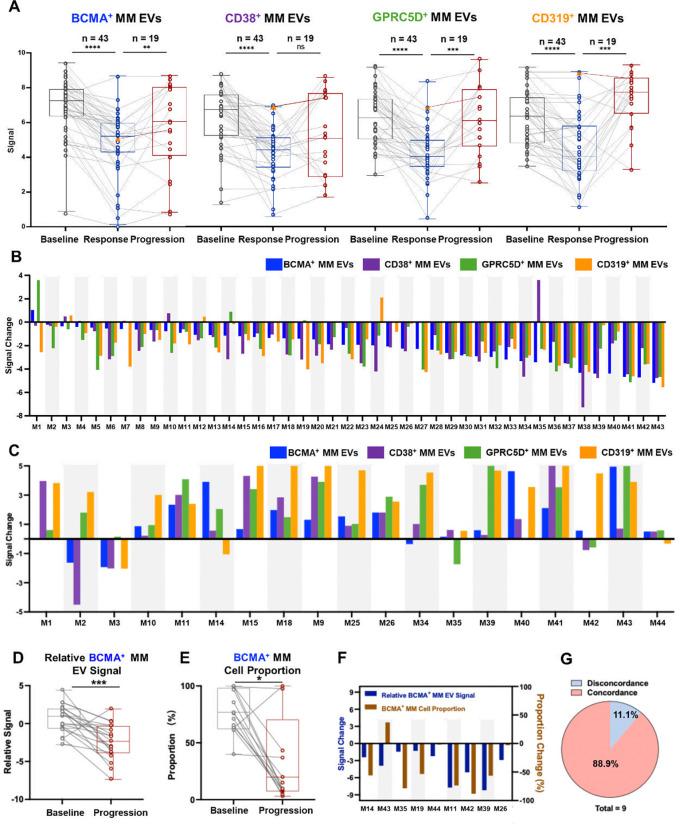
MM EV subpopulations detect treatment response and reflect antigen escape in patients with RRMM following anti-BCMA CAR T-cell therapy. **(A)** Boxplots showing significantly decreased signals in all four MM EV subpopulations (i.e., BCMA^+^, CD38^+^, GPRC5D^+^, and CD319^+^ MM EVs) from baseline to the first documented response, as well as increased signals of four MM EV subpopulations from their initial response or minor response (MR for one case, labeled as orange triangle) to progression. Statistical significance was determined by paired Student’s t-test. **p <0.01, ***p <0.001, ****p <0.001, ns = no significance. **(B)** Waterfall plot showing the signal changes of four MM EV subpopulations at each patient’s first documented response as defined by the IMWG criteria (CR/sCR or PR/VGPR; n = 43). Signal changes represent the differences between signals at the first documented response and those at baseline. **(C)** Waterfall plot documenting the signal changes of four MM EV subpopulations from initial response or MR to first documented progression (progressive disease [PD] or relapse after CR; n = 19), calculated by subtracting signals at initial response or MR from those at first documented progression. **(D)** Boxplot showing decreased signals of relative BCMA^+^ MM EV signals from baseline to first documented progression in 19 patients with PD. Statistical significance was assessed using a paired Student’s t-test (***p <0.01). **(E)** Boxplot showing decreased BCMA^+^ MM cell proportion in bone marrow aspirates from baseline to progression in 9 patients with available data. Statistical significance was compared using the paired Student’s t-test (*p <0.05). **(F)** Waterfall plot comparing changes in BCMA^+^ MM cell proportions (assessed by bone marrow flow cytometry) and relative BCMA^+^ MM EV signals from baseline to progression. **(G)** Pie chart summarizing concordance between EV-based and bone marrow–based assessments of BCMA antigen escape. Among 9 patients with confirmed BCMA antigen escape by decreased BCMA^+^ MM cell proportion, 8 (88.9%) showed concordant decreases in relative BCMA^+^ MM EV signals. BCMA, B-cell maturation antigen; CD, cluster of differentiation; EV, extracellular vesicle; GPRC5D, G protein-coupled receptor family C group 5 member D; MM, multiple myeloma.

As summarized in Fig. 5A, all four MM EV subpopulations consistently declined from baseline to first documented response in 43 patients who achieved a clinical response, with paired t-tests confirming statistically significant decreases across all subpopulations. In 19 patients with progression (18 with an initial clinical response and one with an initial minor response (MR)), paired t-tests demonstrated statistically significant increases in BCMA^+^, GPRC5D^+^, and CD319^+^ MM EVs at progression compared with their initial response (or MR), while CD38^+^ MM EVs also trended upward but did not reach statistical significance. All 43 patients (100%) exhibited decreases with negative signal changes from baseline to first documented response in at least one MM EV subpopulation (Fig. 5B), while all 19 patients (100%) showed increases with positive signal changes from their initial response (or MR for Patient M44) to progression in at least one MM EV subpopulation (Fig. 5C). These results demonstrate that MM EV dynamics robustly capture both treatment response and disease progression, underscoring the complementary value of multiplexed profiling of MM EV subpopulations for real-time disease monitoring in RRMM.

Since BCMA antigen escape in PD cases was clinically determined by flow cytometry using bone marrow samples, we investigated whether the BCMA^+^ MM EVs could serve as a noninvasive surrogate to detect BCMA antigen escape in PD cases. Similar to the calculation of BCMA^+^ MM cell proportion in bone marrow, which defined by normalizing BCMA^+^ MM cells to the total MM cell population (i.e., tumor burden), relative BCMA^+^ MM EV signals were calculated by normalizing BCMA^+^ MM EV signals to CD319^+^ MM EVs, as CD319^+^ MM EVs showed the best performance in reflecting disease status across treatment response and disease progression in PD patients (Fig. 5A). Among the 19 patients who experienced disease progression, 14 (73.7%) showed significantly decreased relative BCMA^+^ MM EV signals at the time of progression compared with baseline (Fig. 5D). In contrast, no significant signal decrease was observed for CD38^+^ MM EVs, and signals for the CD319^+^ MM EVs and GPRC5D^+^ MM EVs even increased at the time of progression compared with baseline (**Fig. S3**). These findings suggest that while overall tumor burden, as reflected by the other three MM EV subpopulations, remained comparable or even increased to baseline, BCMA expression is reduced relative to tumor burden, which indicates antigen escape or even target loss. Of the 14 patients with decreased relative BCMA^+^ MM EV signals, 9 had paired bone marrow samples available for flow cytometric analysis, which confirmed a significant decrease in BCMA^+^ MM cell proportion at progression compared to baseline (Fig. 5E). Among these 9 patients, 8 (88.9%) exhibited concordant decreases in both relative BCMA^+^ MM EV signals and BCMA^+^ MM cell proportion (Fig. 5F–G), supporting the potential of the MM EV Surface Protein Assay as a noninvasive tool to detect antigen escape and inform resistance mechanisms.

### MM EV Subpopulations Complement MRD for Detecting Depth of Response and Early Disease Progression in RRMM

**Fig. 6.**
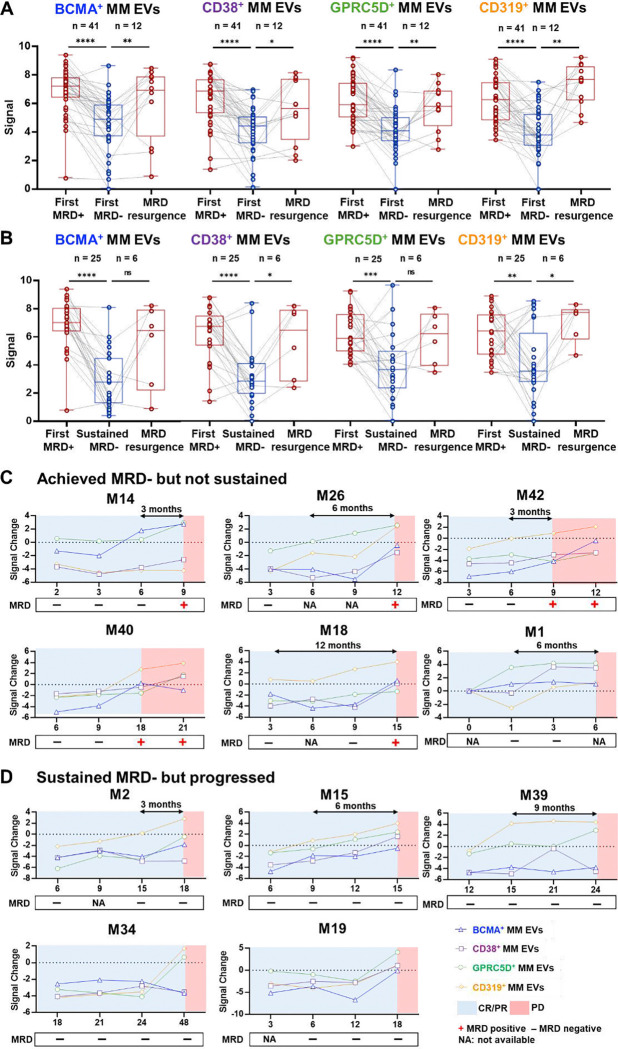
Longitudinal dynamics of MM EV subpopulations in relation to minimal residual disease (MRD) dynamics and clinical outcome. (**A-B**) Paired comparisons of the four MM EV subpopulations across MRD status transitions. Longitudinal trajectories of MM EV subpopulations in patients with progressed disease in subgroups of **(C)** achieved MRD negativity but not sustained, and **(D)** sustained MRD negativity but progressed. MM EV signal changes were computed as the follow-up signal minus baseline signal. Color-coded dots represent EV signals: BCMA^+^ MM EVs (blue), CD38^+^ MM EVs (purple), GPRC5D^+^ MM EVs (green), and CD319^+^ MM EVs (orange). MRD status at each time point is indicated as “+” (positive) or “−” (negative). Shaded regions correspond to the clinical progression phase (red), or response period (blue). Lead time prior to clinical progression is indicated as (⟷), and x-axis indicates time in months after CAR T-cell infusion. BCMA, B-cell maturation antigen; CD, cluster of differentiation; EV, extracellular vesicle; GPRC5D, G protein-coupled receptor family C group 5 member D; MM, multiple myeloma; MRD, minimal residual disease; CR/PR, complete or partial response; PD, progressive disease.

To evaluate whether MM EV subpopulations detect MRD status, we compared the signals of four MM EV subpopulations between MRD-positive and MRD-negative groups. The MRD-positive group included patients with either (i) first MRD positivity at baseline prior to anti-BCMA CAR T-cell therapy or (ii) MRD resurgence after achieving MRD negativity. The MRD-negative group included patients with either (i) first documented MRD negativity following therapy or (ii) sustained MRD negativity, defined as two consecutive MRD-negative results ≥12 months apart. As summarized in Fig. 6A–B, all four EV subpopulations significantly differentiated first MRD positivity from either first MRD negativity or sustained MRD negativity, as well as first MRD negativity from MRD resurgence. Notably, CD38^+^ MM EVs and CD319^+^ MM EVs significantly distinguished sustained MRD negativity from subsequent MRD resurgence. In contrast, BCMA^+^ MM EVs showed a trend toward an increase without achieving statistical significance, and 5 out of the 6 patients who developed MRD resurgence had antigen escape confirmed at disease progression (Fig. 5E–F), potentially reflecting antigen escape also at MRD resurgence. Among 41 patients who achieved MRD negativity, 12 (29.3%) experienced MRD resurgence, including 6 of 25 (24.0%) who had initially reached sustained MRD negativity. By differentiating MRD status, MM EV subpopulations demonstrate potential as biomarkers for detecting depth of response.

To further evaluate the dynamics of MM EV subpopulations and MRD status in monitoring early relapse or disease progression, we analyzed longitudinal data from all 19 RRMM patients who developed disease progression following anti-BCMA CAR T-cell therapy. MM EV signal changes were calculated by subtracting the baseline signal from each follow-up time point, with a positive signal change indicating potential disease progression detected by the assay. Longitudinal trajectories of MM EV subpopulations in 19 patients with progressed disease were displayed and stratified into four MRD dynamic patterns: i) Achieved MRD negativity but not sustained (Fig. 6C), ii) Sustained MRD negativity but progressed (Fig. 6D); iii) MRD resurgence after sustained MRD negativity **(Fig. S4A)**; iv) Never achieved MRD negativity **(Fig. S4B)**. MM EV subpopulations were especially informative in patients with (i) MRD negativity not sustained, where our assay detected progression in all 6 cases and earlier than MRD in 5 out of 6 cases; and (ii) sustained MRD negativity but progression, where EV signals captured progression in all 5 cases despite persistently negative MRD by cytometry at progression. Overall, as summarized in **Table S2**, MM EV subpopulations demonstrated superior sensitivity for early relapse detection compared with MRD. Among the 19 patients with progressive disease, 12 (63.2%) showed positive signal changes in at least one MM EV subpopulation, with a median lead time of 6 months before clinical progression. In contrast, only 3 of 19 patients (15.8%) were identified as relapsing by bone marrow–based MRD, with a shorter median lead time of 1.5 months (χ^2^ = 7.74, p < 0.01). At the time of clinical progression, MM EV subpopulations remained positive in 16 of 19 patients (84.2%), compared with 11 of 17 evaluable cases (64.7%) by EuroFlow cytometry MRD (χ^2^ = 1.54, p = 0.22). These findings suggest that incorporating MM EV subpopulation monitoring alongside conventional MRD assessment could enhance early relapse detection and improve disease progression monitoring in RRMM.

### MM EV Subpopulations as Early Predictors of PFS and OS in RRMM Patients Who Achieved MRD Negativity After CAR T-Cell Therapy

**Fig. 7.**
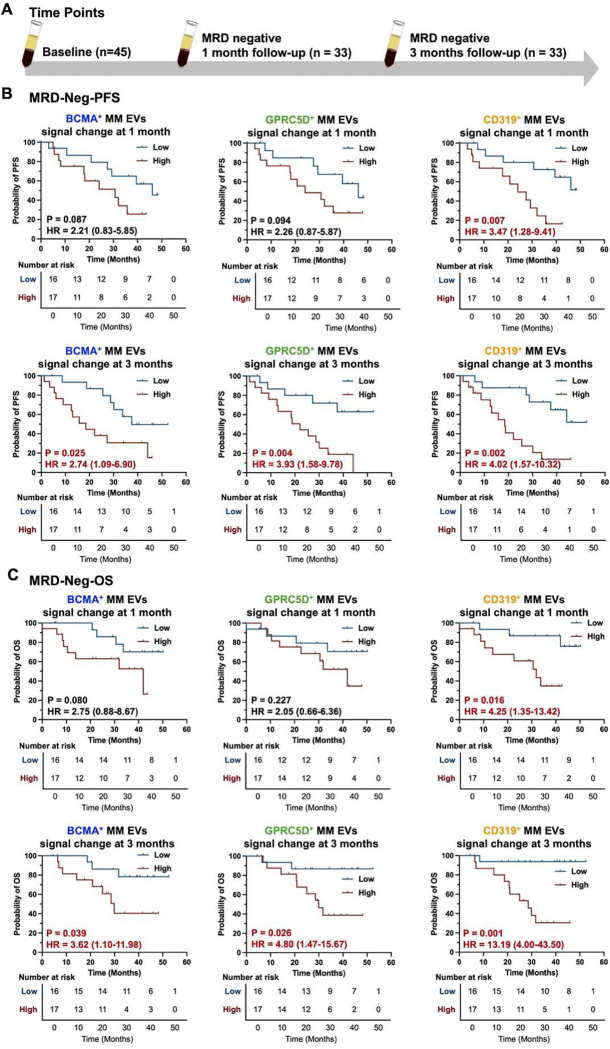
Prognostic value of MM EV subpopulations for predicting progression-free survival and overall survival in MRD-negative RRMM patients after anti-BCMA CAR T-cell therapy. (**A**) Blood samples were collected at different time points. Kaplan-Meier survival curves for (**B**) PFS and (**C**) OS specifically in MRD-negative patients (termed MRD-Neg-PFS and MRD-Neg-OS), stratified by BCMA^+^, GPRC5D^+^ and CD319^+^ MM EVs signal changes at early follow-up time points (1 month and 3 months post-therapy). Patients were grouped as “low” or “high” using the median value as cutoff. Prognostic significance was assessed using log-rank tests, and hazard ratios (HR) with 95% confidence intervals (CI) are provided. Signal changes were calculated as follow-up minus baseline values. BCMA, B-cell maturation antigen; CD, cluster of differentiation; EV, extracellular vesicle; GPRC5D, G protein-coupled receptor family C group 5 member D; MM, multiple myeloma; MRD, minimal residual disease; OS, overall survival; PFS, progression free survival.

It is well established that achieving MRD negativity is associated with longer PFS and OS compared to MRD-positivity.^44^ To further investigate whether the MM EV Subpopulations can stratify survival within the MRD-negative cohort, we evaluated PFS and OS specifically in MRD-negative patients (termed MRD-Neg-PFS and MRD-Neg-OS) following CAR T-cell therapy (Fig. 7A). The longitudinal signal changes of MM EV subpopulations were defined as follow-up subtracting baseline signals and their correlation with MRD-Neg-PFS and MRD-Neg-OS were assessed. Patients were stratified into high or low signal change groups using median value as the cutoff at each follow-up time point. Among the four MM EV subpopulations, CD319^+^ MM EVs were the only biomarker that effectively predicted MRD-Neg-PFS at one month post-treatment. At three months post-treatment, all MM EV subpopulations except CD38^+^ MM EVs were effective in predicting MRD-Neg-PFS (Fig. 7B, **Fig. S5A**). MRD-negative patients with lower signal changes in MM EVs experienced longer MRD-Neg-PFS compared to those with higher signal changes from 1 to 3 months post-treatment. Notably, CD319^+^ MM EVs have significant prognostic value on MRD-Neg-PFS from 1 month to 3 months follow-up with a hazard ratio (HR) ranging from 3.47 to 4.02 by log-rank tests.

A similar trend was observed for MRD-Neg-OS, where higher signal changes in MM EVs correlated with poorer OS (Fig. 7C, **Fig. S5B**). Among the four MM EV subpopulations, CD319^+^ MM EVs were the only biomarker that effectively predicted MRD-Neg-OS at one month post-treatment. At three months post-treatment, all MM EV subpopulations except CD38^+^ MM EVs were effective in predicting MRD-Neg-OS. CD319^+^ MM EVs showed significant prognostic value for MRD-Neg-OS from 1 month to 3 months post-treatment (HR range: 4.25 to 13.19 by log-rank tests). These findings further support that the MM EV subpopulations can predict PFS and OS even within MRD-negative patients. Overall, longitudinal assessment of CD319^+^ MM EVs provides dynamic and significant prognostic value, enabling early prediction (as early as one month) of both PFS and OS following anti-BCMA CAR T-cell therapy in RRMM patients who achieved MRD negativity.

## Discussion

In this study, we developed and applied a novel, noninvasive MM EV Surface Protein Assay for dynamically tracking depth of response, early relapse, and MRD-negative survival outcomes in RRMM patients receiving anti-BCMA CAR T-cell therapy. This assay enables quantitative detection of four MM EV subpopulations, each defined by a clinically actionable surface protein, directly from patient plasma. By integrating the sensitivity of EV-based detection with the clinical relevance of therapeutic targets, the assay addresses limitations of conventional monitoring approaches that rely on invasive bone marrow sampling.

Current assessment of hematologic treatment response of MM primarily relies on the plasma cell count in bone marrow measured by flow cytometry and the M-protein level in blood or urine measured by protein electrophoresis and immunofixation ^20^. Conventional profiling of targetable surface proteins in RRMM typically requires bone marrow–based techniques such as NGF, IHC, or NGS ^22,24^. Despite their effectiveness, these methods are invasive, costly, and not feasible for frequent monitoring, especially given the spatial heterogeneity of MM ^45^. Liquid biopsy approaches, such as ctDNA-based NGS assays, have been developed for detecting MRD ^27,28^, treatment response ^29,30^, and survival outcomes ^31^ in MM. However, these assays are often limited by low tumor fraction, the need for individualized mutation tracking ^22,46^, and they are not capable of directly measuring surface proteins ^22,25^. In contrast, our MM EV Surface Protein Assay provides a cost-effective, noninvasive, and antigen-specific readout for dynamically quantifying multiple clinically actionable surface proteins in parallel without requiring prior bone marrow sequencing. This advantage is particularly valuable for monitoring antigen escape and target evolution. Moreover, the assay is both broadly applicable across patients and adaptable to emerging therapeutic targets.

Importantly, we found that decreases of relative BCMA^+^ MM EV signals reflected potential therapeutic antigen escape following anti-BCMA CAR T-cell therapy. Notably, in progressive disease, BCMA^+^ MM EV levels declined while other MM EV subpopulations (e.g., CD319^+^, GPRC5D^+^ MM EVs) remained elevated, indicating selective BCMA loss rather than disease regression. This supports antigenic escape and highlights the complementary value of multiplexed MM EV profiling as a noninvasive, cost-effective, real-time alternative to bone marrow–based detection, potentially reducing repeated invasive procedures.

Notably, MM EV subpopulations also differentiate MRD status measured by IMWG-recommended bone marrow-based MRD tests, indicating their ability to detect the depth of response defined by MRD negativity or sustained MRD negativity. Our results also suggest that incorporating MM EV subpopulation monitoring alongside current MRD assessment could enhance early relapse detection in RRMM, particularly in patients classified as sustained MRD negativity or those who achieved but failed to sustain MRD negativity. Moreover, in patients with disease progression, at least one of the four MM EV subpopulations consistently exhibited a signal increase above baseline, suggesting that different subpopulations may dominate at different stages of disease evolution. These findings underscore the complementary roles of the MM EV subpopulations to bone marrow-based MRD testing and demonstrate the advantages of multiplexed EV profiling for detecting depth of response, early relapse and disease progression.

Our findings indicate that among the four MM EV subpopulations, CD319^+^ MM EVs may serve as the most robust biomarker in the setting of RRMM patients receiving anti-BCMA CAR T-cell therapy. This aligns with previous studies identifying CD319 as an effective alternative biomarker in the context of anti-CD38 immunotherapy ^47^. This is also clinically meaningful given the emergence of CD319 (SLAMF7/CS1)-targeted strategies such as elotuzumab, which is FDA-approved for use in combination regimens for RRMM ^48,49^. Ongoing clinical trials are also exploring CD319-targeted CAR T-cell therapies and antibody-drug conjugates in advanced MM settings ^50,51^. As the therapeutic landscape continues to evolve, monitoring antigen escape and identifying alternative targetable surface proteins via MM EV Surface Protein Assay may enable adaptive, biomarker-driven treatment planning. Given its scalability and ability to dynamically quantify targetable MM surface proteins, this MM EV Surface Protein Assay holds promise for future clinical companion diagnostic applications.

Despite these promising findings, this study has several limitations. First, as a retrospective, single-center biomarker study, it is subject to inherent limitations in patient selection and data uniformity. Second, the relatively small cohort size limits the statistical power for more detailed subgroup analyses. Future research will involve larger, prospective, multicenter studies to validate the MM EV Surface Protein Assay and confirm its clinical utility in RRMM patients receiving other immunotherapies.

In conclusion, we developed and applied a noninvasive MM EV Surface Protein Assay capable of longitudinally tracking the evolution of four clinically actionable MM surface proteins in RRMM patients receiving anti-BCMA CAR T-cell therapy. This assay effectively captured dynamic changes in four MM EV subpopulations, enabling real-time detection of treatment response, disease progression, and antigen escape. Importantly, MM EV subpopulations emerged as complementary biomarkers to current MRD assessment, enhancing the detection of depth of response, early relapse, and refining survival stratification for both PFS and OS in patients who achieved MRD negativity. This liquid biopsy platform offers translational potential to augment existing clinical diagnostic modalities and guide precision treatment strategies not only in the setting of CAR T-cell therapy, but also in emerging modalities such as antibody–drug conjugates and bispecific antibody therapies in RRMM.

## Methods

### An integrated bioinformatic framework for identifying Targetable MM EV surface proteins

Two datasets were employed for the selection of overall MM EV-specific surface proteins: a) Clinicaltrials.gov which identified 60 targetable MM proteins. We performed an exhaustive search and rigorous manual curation to compile therapeutic targets of antibody-based treatments, including antibody-drug conjugates (ADCs), CAR-T therapies, monoclonal antibodies (mAbs), and bispecific antibodies. b) The Pharos database (https://pharos.nih.gov), which initially identified 1,437 targetable MM proteins. Target Development Levels “Tclin” and “Tchem,” representing clinically validated and preclinical targets respectively, were used to refine the list, narrowing it down to 285 targetable MM proteins. Subsequently, MM EV-specific surface proteins were identified through The Cancer Surfaceome Atlas (TCSA) data ^52^ (GESP score > 8) and Vesiclepedia ^53,54^ (EV detection frequency) or AmiGO2 data (https://amigo.geneontology.org/) based on Gene Ontology term (“extracellular vesicle/ extracellular exosome”). Following this selection, the MM-specific surface proteins were further refined by analyzing their expression levels using Differentiation Map (DMAP) data ^55^ and proteome profile data ^56^, where proteins with high median expression in immune cells (DMAP ≥ 7) and expression less than 0 in MM proteomic profile were excluded to minimize background noise. The top four proteins with the highest RNA expression levels from the GDC MMRF-COMMPASS RNA data (downloaded from the UCSC Xenabrowser, https://xena.ucsc.edu/) were selected, resulting in four MM EV surface proteins: CD38, CD319, BCMA, and GPRC5D.

### MM Cell Line Culture and MM EVs Collection from Cell Culture Medium

MM cell line MM.1S was obtained from Shanghai Institute of Biochemistry and Cell Biology (SIBCB) (Shanghai, China). The MM.1S cell line was cultured in respective Iscove’s Modified Dulbecco’s Medium (IMDM, Thermo Fisher Scientific, USA) and Eagle’s Minimum Essential Medium (EMEM, Thermo Fisher Scientific, USA), supplemented with 10% fetal bovine serum (FBS) and 100 U mL^−1^ penicillin-streptomycin (Thermo Fisher Scientific, USA). Immunofluorescence (IF) staining was performed according to our previous study ^41^.

To collect MM cell-derived EVs, MM.1S cells were grown to 80% confluence in 18 Nunc EasYFlask Flasks (175 cm^2^, Thermo Fisher Scientific, USA). The culture medium was then replaced with serum-free medium (13 mL per flask) to induce cell starvation for 24 h. The conditioned medium was collected and centrifuged at 300 g for 10 min, followed by a second centrifugation at 2800 g for 10 min to remove cell debris. The medium was subsequently ultracentrifuged at 100 000 g for 90 min, and the EV pellet was resuspended in 400 μL PBS.

### Synthesis of EV Click Beads

The EV Click Beads (10 mg, 6 × 10^8^ beads, 2.5 μm in diameter, and 2.0 g cm^−3^ density) were first incubated in 2.0 N nitric acid (HNO_3_) for 10 min to regenerate hydroxyl groups. Subsequently, they were silanized in an ethanol solution containing 4% v/v (3-aminopropyl) triethoxysilane (APTES, 25 μL) for 45 min at room temperature. The amino-functionalized silica microbeads were washed with ethanol to remove unbound silane and then reacted with mTz-PEG-NHS ester (0.94 mg, 3.8 mm) in DMSO/PBS (pH = 8.4, 600 μL) for 60 min at room temperature.

### Preparation of TCO-Grafted Antibodies

The TCO-grafted CD38, CD319, BCMA, and GPRC5D antibodies were synthesized by incubating TCO-PEG4-NHS ester (4 μm, Click Chemistry Tools) with antibodies (1 mg mL^−1^, 20 μL) in BBS (pH = 8.2) for 30 min at room temperature. The resulting TCO-antibody conjugates were aliquoted and stored at −20 °C until use.

### Characterization of MM EVs

Nanoparticle tracking analysis (NTA) was performed using a ZetaView PMX-120 (Particle-Metrix, Germany) to determine the size distribution and concentration of MM.1S EVs. Samples were diluted in 0.22 μm filtered PBS at appropriate dilution rates ranging from 100 to 10 000-fold. Each sample was analyzed in triplicate. For electron microscopy, EV samples were fixed in 4% PFA for 30 min and prepared for transmission electron microscopy (TEM). For immunogold staining, fixed EV-bound beads were incubated with monoclonal anti-human CD63 mouse IgG antibody (Abcam, 1:50 dilution), followed by antimouse nanogold (10 nm, 1:20 dilution), then prepared for TEM imaging.

### Patient enrollment

In this retrospective, single-center study, patients with RRMM who received anti-BCMA CAR T-cell therapy at Tongji Hospital between May 2020 and August 2024 were included. Both commercial products (Equecabtagene Autoleucel and Zevorcabtagene Autoleucel) and academic, point-of-care CAR T-cell products were evaluated. Detailed inclusion and exclusion criteria are provided in **Supplementary Methods**. The study was approved by the Ethics Committee of Tongji Hospital, Tongji Medical College, Huazhong University of Science and Technology (TJIRB202406069). All patients received lymphodepletion with fludarabine (25 mg/m^2^/day) and cyclophosphamide (20 mg/kg/day) over three consecutive days (days −4 to −2) prior to CAR T-cell infusion. Prior to application in the RRMM cohort, the MM EV Surface Protein Assay was validated in an independent analytical cohort of treatment-naïve MM patients and healthy donors (HDs) at UCLA. These treatment-naïve MM samples were purchased from commercial biobanks Proteogenex (USA), and HDs were recruited at UCLA under an IRB-approved protocol (UCLA IRB#19–000857). All procedures were conducted in accordance with the Declaration of Helsinki.

### Inclusion and exclusion criteria

Eligible patients were required to be between 18 and 70 years of age, with a documented diagnosis of multiple myeloma based on International Myeloma Working Group (IMWG) criteria^57^. All had received at least three prior lines of therapy, including a proteasome inhibitor and an immunomodulatory agent, and had experienced disease progression either during treatment or within 12 months after their most recent anti-myeloma therapy. For patients whose most recent treatment was CAR T-cell therapy, progression was not restricted to the 12-month timeframe. Evidence of BCMA expression on tumor cells, confirmed by immunohistochemistry or flow cytometry, was required for enrollment. Key exclusion criteria included a history of graft-versus-host disease (GVHD) or the need for long-term immunosuppressive therapy. Patients were excluded if they had received autologous hematopoietic stem cell transplantation (auto-HSCT) within 12 weeks prior to apheresis, had undergone two prior auto-HSCTs, or had a history of allogeneic HSCT. Patients were also excluded if peripheral blood mononuclear cells were insufficient for CAR T-cell manufacturing.

### Sample collection and processing

Blood samples were collected from each patient prior to anti-BCMA CAR T-cell infusion and at each subsequent follow-up visit for up to 48 months. Peripheral blood was drawn into EDTA-containing BD Vacutainer tubes (Cat. #366643) and processed within 4 hours after collection. Plasma was isolated via centrifugation at 530 g for 10 minutes, followed by 4,600 g for 10 minutes to remove cellular debris. Samples were aliquoted and stored at −80°C until analysis.

### Preparation of Synthetic Plasma Samples

For synthetic plasma samples in linearity study of MM EV Surface Protein Assay, 10 μL aliquots of MM EV pellets were added to 90 μL of EV-depleted plasma from male healthy donors, with serial dilutions of the spiked MM.1S EVs. 100 μL synthetic plasma or clinical plasma samples were mixed with one of the four TCO-grafted MM EV-specific antibodies for 45 min. These samples were incubated with EV Click Beads, followed by centrifugation and washing, and then the four subpopulations of MM EVs (i.e., CD38^+^ MM EVs, BCMA^+^ MM EVs, GPRC5D+ MM EVs, and CD319^+^ MM EVs) were subjected to RT-qPCR for quantification. Prior to testing clinical samples, synthetic MM plasma samples using spiked MM.1S EVs into HD plasma were employed to determine the performance of EV Click Beads.

### MM EV Surface Protein Assay

The MM EV surface protein assay consisted of two steps: i) click chemistry-mediated enrichment of MM EVs by EV Click Beads in the presence of one of the four trans-cyclooctene (TCO)-grafted antibodies, and ii) quantification of enriched MM EVs by reverse transcription-quantitative polymerase chain reaction (RT-qPCR) detecting the *ACTB* mRNA. In brief, 100 ng of trans-cyclooctene (TCO)-conjugated antibody (anti-BCMA, anti-CD38, anti-GPRC5D, or anti-CD319) was added to 100 μL of plasma and incubated at room temperature for 45 minutes. Antibody-labeled EVs were then incubated with 50 μg of EV Click Beads for 45 minutes, followed by centrifugation and washing steps. EV-enriched beads were lysed using XpressAmp Lysis Buffer with 1% thioglycerol (Promega). Reverse transcription–quantitative PCR (RT-qPCR) was performed using PrimeDirect Probe RT-qPCR Mix (Takara) targeting *ACTB* mRNA, and analyzed using a CFX Duet Real-Time PCR System (Bio-Rad). The signal is represented as 40 – Ct value. To minimize bias, the study was conducted under a blinded workflow: clinical researchers collecting samples were blinded to experimental results, and investigators conducting EV assays were blinded to clinical outcomes.

### Outcome assessment

Clinical efficacy was evaluated according to the International Myeloma Working Group (IMWG) consensus criteria ^57^. Outcomes included progression-free survival (PFS) and OS. PFS was measured from the date of blood sample collection to documented disease progression or death from any cause; OS was defined as the time from sample collection to death or last follow-up.

For patients with extramedullary disease (EMD), complete response (CR) was assessed using bone marrow aspirate and biopsy, serum and urine M-protein quantification, and imaging ^58^. MRD was evaluated by EuroFlow cytometry during bone marrow aspiration, with a sensitivity threshold of 10^−5^ nucleated cells. Disease staging was determined by the Revised International Staging System (R-ISS), incorporating baseline International Staging System stage, cytogenetic abnormalities, and serum lactate dehydrogenase (LDH) levels. High-risk cytogenetics was defined by the presence of del(17p), t(4;14), or t(14;16), as detected by fluorescence in situ hybridization (FISH).

### Statistical Analysis

Categorical variables were analyzed using the χ^2^ test or Fisher’s exact test, and continuous variables were compared using Student’s t-test or paired t-test. Differences in the slopes of the regression lines were assessed using Wald t-tests. PFS and OS were evaluated using the Kaplan-Meier method, and 95% confidence intervals (CIs) were reported. Statistical analyses were conducted using SPSS version 22, GraphPad Prism version 9 and R Studio (version 4.2.3). A two-tailed P value ≤ 0.05 was considered statistically significant.

## Supplementary Material

Supplementary Files

This is a list of supplementary files associated with this preprint. Click to download.

• SupplementaryMaterialsNC.pdf

## Figures and Tables

**Table 1.** Patient characteristics

Patient characteristics	Total (n=45)

Age, y; median (IQR)	57 (53–61)
Sex, n (%)	
Female	23 (51.1)
Male	22 (48.9)
Extramedullary disease^a^, No. (%)	7 (15.6)
Myeloma subtype, n (%)	
IgG	21 (46.7)
IgA	8 (17.8)
IgD	3 (6.7)
Kappa	5 (11.1)
Lambda	7 (15.6)
Non secretory	1 (2.2)
Durie-Salmon stage, n (%)	
I	5 (11.1)
II	4 (8.9)
III	36 (80.0)
R-ISS^b^, n (%)	
I	7 (16.3)
II	30 (69.8)
III	6 (13.9)
High-risk cytogenetics^c^, n (%)	
0	29 (67.4)
1	10 (23.3)
2	4 (9.3)
Triple-exposure^d^, n (%)	12 (26.7)
Penta-exposure^e^, n (%)	10 (22.2)
Previous autologous stem cell transplantation, n (%)	12 (26.7)
Previous CAR-T therapy, n (%)	2 (4.4)
Bridging therapy^f^, n (%)	21 (46.7)
BCMA expression on plasma cells at baseline, median (IQR)	82.9 (63.1–95.4)
BCMA CAR-T products, n (%)	
Equecabtagene Autoleucel	41 (91.1)
Academic CAR-T cells	3 (6.7)
Zevorcabtagene Autoleucel	1 (2.2)
CRS, n (%)	
0–1	39 (86.7)
2–4	6 (13.3)
ICANS, n (%)	0 (0.0)

BCMA, B-Cell Maturation Antigen; CAR, Chimeric Antigen Receptor; CRS, Cytokine Release Syndrome; EMMR-ISS, Revised International Staging System; DS, Durie-Salmon; ICANS, Immune Effector Cell-Associated Neurotoxicity Syndrome; IQR, Interquartile Range.

a Extramedullary disease was defined as a soft tissue mass spreading outside the bone marrow.

b R-ISS disease stage at enrollment was derived from the ISS stage, cytogenetic abnormality (yes vs no), and serum lactate dehydrogenase concentration. Because of the inability to obtain adequate bone marrow specimens from 2 individuals, fluorescence in situ hybridization could not be performed, precluding the analysis.

c Defined as at least 1 del(17p), t(4:14), or t(14:16) detected by fluorescence in situ hybridization. 2 patients did not undergo testing due to an insufficient sample size, and only 43 patients were tested.

d Triple-refractory disease was refractory to immunomodulatory agents, proteasome inhibitors, and monoclonal antibodies.

e Penta-refractory disease was refractory to 2 immunomodulatory agents, 2 proteasome inhibitors, and a monoclonal antibody.

f Therapy was used as a bridge from leukapheresis to lymphodepletion.

## Data Availability

The reagents employed in this investigation are comprehensively presented in this manuscript. Further elucidation on the protocols and statistical code employed in this study can be obtained from the corresponding authors upon request.
